# Vacuolar nitrate efflux requires multiple functional redundant nitrate transporter in *Arabidopsis thaliana*

**DOI:** 10.3389/fpls.2022.926809

**Published:** 2022-07-22

**Authors:** Yu-Ting Lu, De-Fen Liu, Ting-Ting Wen, Zi-Jun Fang, Si-Ying Chen, Hui Li, Ji-Ming Gong

**Affiliations:** ^1^National Key Laboratory of Plant Molecular Genetics, Center for Excellence in Molecular Plant Sciences, Shanghai Institute of Plant Physiology and Ecology, Chinese Academy of Sciences, Shanghai, China; ^2^University of Chinese Academy of Sciences, Beijing, China

**Keywords:** vacuolar nitrate, reallocation, nitrate transporter, osmotic stress, *Arabidopsis thaliana*

## Abstract

Nitrate in plants is preferentially stored in vacuoles; however, how vacuolar nitrate is reallocated and to which biological process(es) it might contribute remain largely elusive. In this study, we functionally characterized three nitrate transporters NPF5.10, NPF5.14, and NPF8.5 that are tonoplast-localized. Ectopic expression in *Xenopus laevis* oocytes revealed that they mediate low-affinity nitrate transport. Histochemical analysis showed that these transporters were expressed preferentially in pericycle and xylem parenchyma cells. *NPF5.10*, *NPF5.14*, and *NPF8.5* overexpression significantly decreased vacuolar nitrate contents and nitrate accumulation in *Arabidopsis* shoots. Further analysis showed that the sextuple mutant (*npf5.10 npf5.14 npf8.5 npf5.11 npf5.12 npf5.16*) had a higher ^15^NO3-uptake rate than the wild-type Col-0, but no significant difference was observed for nitrate accumulation between them. The septuple mutant (*npf5.11 npf5.12 npf5.16 npf5.10 npf5.14 npf8.5 clca*) generated by using CRISPR/Cas9 showed essentially decreased nitrate reallocation compared to wild type when exposed to nitrate starvation, though no further decrease was observed when compared to *clca*. Notably, *NPF5.10*, *NPF5.14*, and *NPF8.5* as well as *NPF5.11*, *NPF5.12,* and *NPF5.16* were consistently induced by mannitol, and more nitrate was detected in the sextuple mutant than in the wild type after mannitol treatment. These observations suggest that vacuolar nitrate efflux is regulated by several functional redundant nitrate transporters, and the reallocation might contribute to osmotic stress response other than mineral nutrition.

## Introduction

Nitrate is the primary nitrogen source and an essential macronutrient for plant growth in well-aerated soil (Crawford and Forde, 2002). Nitrate serves not only as a nitrogen nutrient but also as a signaling molecule (Wang et al., 2018; Hu et al., 2019). Other studies also showed that nitrate acts as an osmotic solute (Smirnoff and Stewart, 1985; Song et al., 2006; Ozfidan-Konakci et al., 2020; Chu et al., 2021; Ozturk et al., 2021). To maintain a stable cytoplasmic nitrate concentration, plants store the excess nitrate in vacuoles. It has been demonstrated that vacuoles are major NO_3_^−^ storage pool and over 90% of cellular nitrate accumulate in there (Martinoia et al., 1981; Granstedt and Huffaker, 1982; Zhen et al., 1991; Miller and Smith, 1996). Several studies proposed that vacuolar nitrate plays an essential role in nutrient storage, osmotic adjustment, and charge balance (Martinoia et al., 1981; Granstedt and Huffaker, 1982; Smirnoff and Stewart, 1985; Steingrover et al., 1986; Zhen et al., 1991; van der Leij et al., 1998), though how it functions remains largely unknown. Since vacuoles do not contain nitrate reductase, proper function for vacuolar nitrate requires it to be released into the cytoplasm when necessary (Martinoia et al., 1981; Rufty et al., 1986; van der Leij et al., 1998; Lea et al., 2004; Cookson et al., 2005).

Up to date, four gene families including NRT1/PTR, NRT2, CLC, and SLAC1/SLAH have been identified in nitrate transport (Lee et al., 2009; Barbier-Brygoo et al., 2011; Dechorgnat et al., 2011; Geiger et al., 2011; Wang et al., 2012, 2018). CHL1, NRT1.2, NRT2.1, NRT2.2, and NRT2.4 are major players in nitrate uptake (Tsay, 1993; Huang et al., 1996, 1999; Wang et al., 1998; Liu et al., 1999; Cerezo et al., 2001; Li et al., 2007; Kiba et al., 2012), while NRT1.5, NRT1.8, and NRT1.9 contribute more to nitrate long-distance transport between roots and shoots (Lin et al., 2008; Li et al., 2010; Wang and Tsay, 2011). For nitrate storage in vacuoles, two transporters have been identified. AtCLCa is tonoplast-localized and mediates 2NO_3_^−^/H^+^ antiport (De Angeli et al., 2006). Though the *clca-1* exhibits normal development and is morphologically indistinguishable from the wild-type control, its capacity to accumulate nitrate is reduced ~50% (Geelen et al., 2000). AtNRT2.7 is another tonoplast localized nitrate transporter and regulates the seed germination by mediating nitrate accumulation in seed vacuoles (Chopin et al., 2007). A previous QTL analysis for shoot nitrate accumulation suggested that AtCLCc might also involve in vacuolar nitrate storage (Harada et al., 2004), though another study proposed that AtCLCc is essential for stomatal movement and salt tolerance by regulating chloride homeostasis (Martinoia et al., 2007; Jossier et al., 2010).

As for vacuolar nitrate efflux, however, the underlying mechanisms remain largely unclear. AtCLCb and OsNPF7.2 are tonoplast-localized nitrate transporters, but disruption of these genes did not affect nitrate accumulation in vacuoles (von der Fecht-Bartenbach et al., 2010; Hu et al., 2016). AtCLCa might also get involved in vacuolar nitrate efflux, as it was proposed to mediate anion release during stomatal closure in response to the stress hormone abscisic acid (ABA; Wege et al., 2014), and nitrate is one of the anions contributing to stomatal movement (Humble and Hsiao, 1969; Guo et al., 2003). Our previous study showed that NPF5.11, NPF5.12, and NPF5.16 were localized to tonoplast and indeed mediated nitrate uptake in oocytes (He et al., 2017); however, only very subtle nitrate accumulation phenotype were observed in the triple mutant. All these observations suggest that vacuolar nitrate efflux might be a more complicated process than expected, or it might conditionally function under certain circumstances.

In this study, we identified three more nitrate transporters NPF5.10, NPF5.14, and NPF.8.5 that localize to the tonoplast. Our research demonstrated that vacuolar nitrate efflux is indeed actively mediated but by multiple functional redundant genes. Both NPF5.10, NPF5.14, NPF8.5, NPF5.11, NPF5.12, and NPF5.16 and AtCLCa contribute to this process and AtCLCa might be the primary one. We further revealed that these transporters might function under osmotic stress.

## Materials and methods

### Plant material and growth conditions

*Arabidopsis thaliana* ecotype Columbia (Col-0) was used in this study. The mutant (*clca*), quadruple mutant (*npf5.11 npf5.12 npf5.16 npf5.10*), quintuple mutant (*npf5.11 npf5.12 npf5.16 npf5.10 npf5.14*), sextuple mutant (*npf5.11 npf5.12 npf5.16 npf5.10 npf5.14 npf8.5*), and septuple mutant (*npf5.11 npf5.12 npf5.16 npf5.10 npf5.14 npf8.5 clca*) were generated using a CRISPR/Cas9 system. The specific guide RNA sequence was introduced into the CRISPR-Cas9 construct (Mao et al., 2013), then the resulted 1,300-bp fragment of Cas9 and specific guide RNA expression cassettes were recovered by *Hind*III/*Eco*RI and subcloned into pCAMBIA1300. To generate transgenic lines overexpressing *NPF5.10*, *NPF5.14* and *NPF8.5*, the CDS sequences of *NPF5.10*, *NPF5.14*, and *NPF8.5* were cloned and fused in frame at the 5′ end of EGFP in the vector 35S:EGFP/pCAMBIA1300. Primer sequences are listed in Supplementary Table S1.

*Arabidopsis* plants were grown in hydroponic solution (4 mM KNO_3_, 0.625 mM KH_2_PO_4_, 0.5 mM MgSO_4_, 0.5 mM Ca(NO_3_)_2_, 0.025 mM Fe-EDTA, 17.5 μM H_3_BO_3_, 3.5 μM MnCl_2_, 2.5 μM NaCl, 0.25 μM ZnSO4, 0.125 μM CuSO_4_, 0.05 μM NaMoO4, 0.0025 μM CoCl_2_, 0.05% (w/v) MES, adjusted to pH 5.7 with KOH) at 22°C with 16-h-light/8-h-dark cycles (Gong et al., 2004). Plants were grown to 4 weeks and then were treated with nitrogen-starved nutrient solution (KNO_3_ and Ca(NO_3_)_2_ were replaced with KCl and CaCl_2_) or 300 mM mannitol as indicated time.

For osmotic stress tolerance assay, surface-sterilized seeds were plated on 1/2 Murashige and Skoog (MS) medium with or without 150 mM mannitol. Plates were kept at 4°C for 2 days, then plants were grown vertically at 22°C with 16-h-light/8-h-dark cycles for 7 days.

### Subcellular localization in plants

CDS sequences of *NPF5.10*, *NPF5.14*, and *NPF8.5* were cloned and fused in frame at the 5′ end of EYFP in the vector 35S:EYFP/PA7. Then, the EYFP fusion plasmids were transformed into protoplasts by polyethylene glycol-mediated transformation (Yoo et al., 2007). The transformed protoplasts were held in the dark at 22°C for more than 30 h. Then, the EYFP fluorescence was imaged using confocal microscopy (ZEISS LSM880).

Overexpression lines harboring *NPF5.10*, *NPF5.14*, and *NPF8.5* were grown for 4 days, then the fluorescence of EGFP in transgenic plants was observed using confocal microscope (LEICA TCS SP8 STED). Primer sequences are listed in Supplementary Table S1.

### Subcellular localization in *Xenopus laevis* oocytes

For the EYFP or EGFP fusion proteins expression in oocytes assay, the constructs as indicated were generated by introducing *NPF5.10* and *NPF5.14* into the vector EYFP-pOO2 using the restriction enzyme *Bam*HI and *Spe*I, and *NPF8.5* were cloned into the vector EGFP-pOO2. Primer sequences are listed in Supplementary Table S1. The cRNA of target genes and positive control NPF7.2 were synthesized using the Ambion mMessage mMachine kit and injected into oocytes as described (He et al., 2017). The fluorescence was imaged using confocal microscope (ZEISS LSM880).

### Transport activity assay in *Xenopus laevis* oocytes

*NPF5.10*, *NPF5.14*, and *NPF8.5* cDNA were cloned into the oocyte expression vector pOO2 (Ludewig et al., 2002). The cRNA was synthesized, and oocytes were injected with 50 ng cRNA or H_2_O, as described previously (Hsu and Tsay, 2013). Oocytes were incubated in ND96 solution for 2 days at 16°C as described (Li et al., 2010). Current recordings were initiated in a bath solution containing 230 mM mannitol, 0.15 mM CaCl_2_, and 10 mM MES/Tris, pH 5.5 (Huang et al., 1999). ^15^NO_3_^−^ uptake/efflux or uptake kinetics assays were performed as described (Huang et al., 1999; Wang and Tsay, 2011; Leran et al., 2013) and the ^15^N content was determined using a continuous-flow isotope ratio mass spectrometer (DELTA V Advantage + Flash 2000). Primer sequences are listed in Supplementary Table S1.

### Histochemical analysis of GUS expression

For GUS staining, the tissues were incubated in a solution containing 50 mM sodium phosphate buffer (pH 7.2), 5 mM K_3_Fe(CN)_6_, 5 mM K_4_Fe(CN)_6_, 0.2% Triton X-100, and 2 mM X-Gluc at 37°C. Semithin sections (6 μm) were imaged using a camera (Nikon). Primer sequences are listed in Supplementary Table S1.

### Quantitative real-time PCR analysis

Plant tissues were sampled as indicated and total RNA was extracted using TRIzol reagent (Invitrogen), then the cDNA was synthesized using PrimeScript TM RT reagent Kit with gDNA Eraser (Takara). Quantitative real-time PCR was performed using a SYBR Premix Ex-Taq (Takara). The *Arabidopsis AtActin2* gene was used as an internal control. Primer sequences are listed in Supplementary Table S1.

### Nitrate content determination by HPLC

Four-week-old hydroponically grown plants were treated as indicated in figure legends. Leaves and roots were harvested and washed at least four times by ultrapure water for 5 min. Or xylem sap was collected as previously described (Sunarpi et al., 2005). Nitrate in samples was determined by HPLC (Agilent 1200 series) using a PARTISIL 10 strong anion exchanger column (Whatman) as described (Chiu et al., 2004).

### Vacuole isolation and nitrate measurement

Rosette leaves from hydroponically grown plants were used to isolate intact protoplasts as described (Yoo et al., 2007), then vacuoles were isolated as described with minor modification (Robert et al., 2007): scaling up the system by a factor of 1.3 and centrifuging it using an Optima L-80XP ultra-centrifuge. Acid phosphatase (ACP) activity specific to vacuole was determined and used to normalize vacuolar nitrate accumulation level.

### Nitrate uptake assay in plants

Wild-type and sextuple mutant plants were grown hydroponically for 21 days and then were transferred to 0.1 mM CaSO_4_ for 1 min, hydroponic culture containing 5 mM K^15^NO_3_ with 99% atom excess of ^15^N for 30 min. At the end of labeling, plants were again transferred to 0.1 mM CaSO_4_ for 1 min, then the shoots and roots were separated and washed at least four times by ultrapure water. The ^15^N content was determined using a continuous-flow isotope ratio mass spectrometer (DELTA V Advantage + Flash 2000).

## Results

### NPF5.10, NPF5.14, and NPF8.5 are localized to the tonoplast

**Figure 1:**
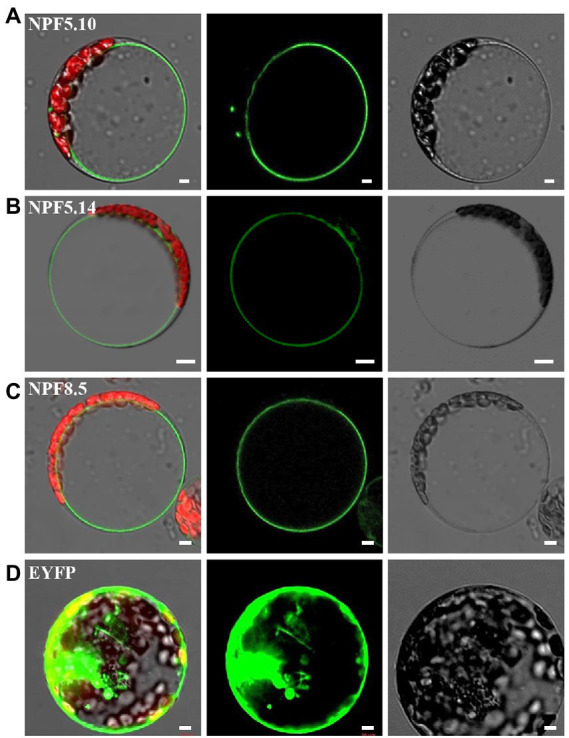
NPF5.10, NPF5.14, and NPF8.5 are tonoplast localized. Expression of *p35S:NPF5.10-EYFP* **(A)**, *p35S:NPF5.14-EYFP* **(B)**, *p35S:NPF8.5-EYFP* **(C),** and *p35S:EYFP* **(D)** in *Arabidopsis* mesophyll protoplasts. The green signals indicate EYFP, and the red signals indicate autofluorescence of chlorophyll. Scale bar, 5 μm.

Our previous studies proposed that NPF5.11, NPF5.12, and NPF5.16 are responsible for vacuolar nitrate efflux in *Arabidopsis*, though significant alteration in vacuolar nitrate content was not detected between the triple mutant and its wild type (He et al., 2017), which raised a question of whether more transporters or channels are involved in mediating vacuolar nitrate release. To answer the question and actively modulate vacuolar nitrate reallocation, we then isolated three other NRT1/NPF family members NPF5.10, NPF5.14, and NPF8.5 according to bioinformatic prediction of their putative subcellular localization.^1^ Subcellular localization assay showed that NPF5.10, NPF5.14, and NPF8.5 were indeed localized to tonoplast in *Arabidopsis* mesophyll protoplasts (Figure 1). Consistent subcellular localization was observed in transgenic plants harboring the construct *p35S:NPF5.10-EYFP*, *p35S:NPF5.14-EYFP* or *p35S:NPF8.5-EYFP* (Supplementary Figure S1).

### NPF5.10, NPF5.14, and NPF8.5 mediate pH-dependent low-affinity nitrate transport

**Figure 2:**
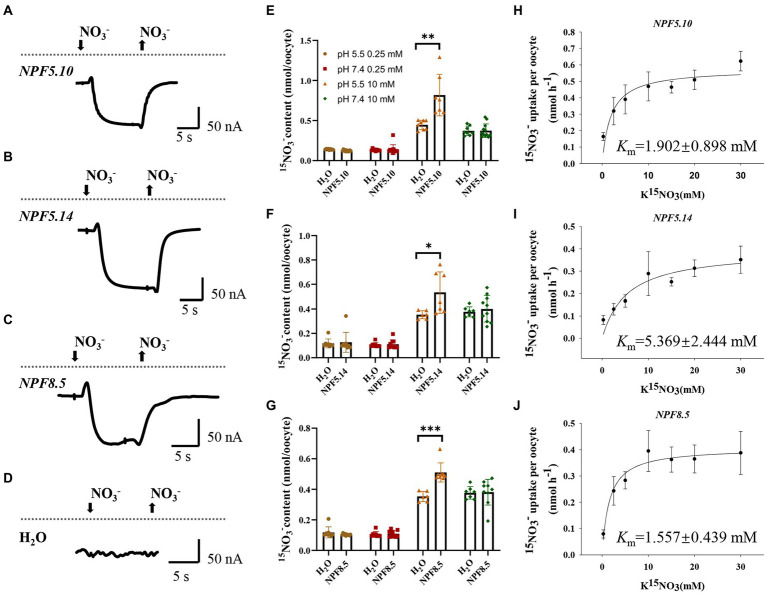
NPF5.10, NPF5.14, and NPF8.5 mediate low affinity nitrate uptake. Oocytes injected with H_2_O, or cRNA of *NPF5.10*, *NPF5.14*, and *NPF8.5* were incubated in ND96 solution for 2 days at 16°C. **(A–D)** The oocytes were then voltage clamped at −60 mV and representative inward currents were recorded under 10 mM NO_3_^−^, pH 5.5 treatment. **(E–G)** Oocytes were transferred into solution containing 0.25 mM ^15^NO_3_^−^ or 10 mM ^15^NO_3_^−^, incubated in buffer at either pH 5.5 or 7.4 for 6 h. Values are mean ± SD, *n* = 8–12 oocytes. Statistical significance was determined by Student’s *t*-test (^*^p < 0.05; ^**^p < 0.01; ^***^p < 0.001). **(H–J)** Oocytes injected with *NPF5.10*, *NPF5.14*, or *NPF8.5* cRNA were incubated with different concentrations of K^15^NO_3_ at pH 5.5 for 1.5 h, then their ^15^N content was determined and the *K*_m_ was calculated. Values are mean ± SD, *n* = 8–12 oocytes.

Given *X. laevis* oocytes do not have vacuoles, we firstly tested the expression and localization of NPF5.10, NPF5.14, and NPF8.5 in oocytes, as described in the article (He et al., 2017), and the result indicated that the NPF5.10-EYFP, NPF5.14-EYFP, and NPF8.5-EGFP fusion proteins expressed in plasma membrane of oocytes (Supplementary Figure S2). As the cytosolic side of tonoplast protein should remain at the cytosolic side in oocytes (He et al., 2017; Xu et al., 2019), nitrate transport activities of NPF5.10, NPF5.14, and NPF8.5 were then measured by electrophysiological analysis using cRNA-injected oocytes. Inward currents were elicited by 10 mM nitrate at pH 5.5 in *NPF5.10*, *NPF5.14*, and *NPF8.5* cRNA-injected oocytes, suggesting that they did mediate low affinity nitrate transport at pH 5.5 (Figures 2A–D).

Further nitrate uptake assay was performed using ^15^NO_3_^−^. Compared with water-injected oocytes, the *NPF5.10*, *NPF5.14*, and *NPF8.5* cRNA-injected oocytes showed significantly enhanced ^15^NO_3_^−^ uptake when incubated with 10 mM ^15^NO_3_^−^ at pH 5.5 (Figures 2E–G), in contrast to other conditions including 0.25 mM ^15^NO_3_^−^ at pH 5.5/7.4 and 10 mM ^15^NO_3_^−^ at pH 7.4. In addition, we did not observe nitrate efflux from oocytes when incubated in ND96 solution at pH 5.5 (Supplementary Figure S3), suggesting that NPF5.10, NPF5.14, and NPF8.5 regulate nitrate transport unidirectionally. The *K*_m_ values of *NPF5.10*, *NPF5.14,* and *NPF8.5* for nitrate was further calculated using different concentrations of ^15^NO_3_^−^ ranging from 0.25 mM to 30 mM. A Michaelis–Menten analysis showed that the *K*_m_ values of NPF5.10, NPF5.14, and NPF8.5 were ~1.902, 5.369, and 1.557 mM, respectively (Figures 2H–J). These data together demonstrate that NPF5.10, NPF5.14, and NPF8.5 mediate pH-dependent low-affinity nitrate uptake into cytosol. In other words, they should mediate vacuolar nitrate efflux into cytosol in plants.

### *NPF5.10*, *NPF5.14*, and *NPF8.5* are preferentially expressed in vascular tissues

**Figure 3:**
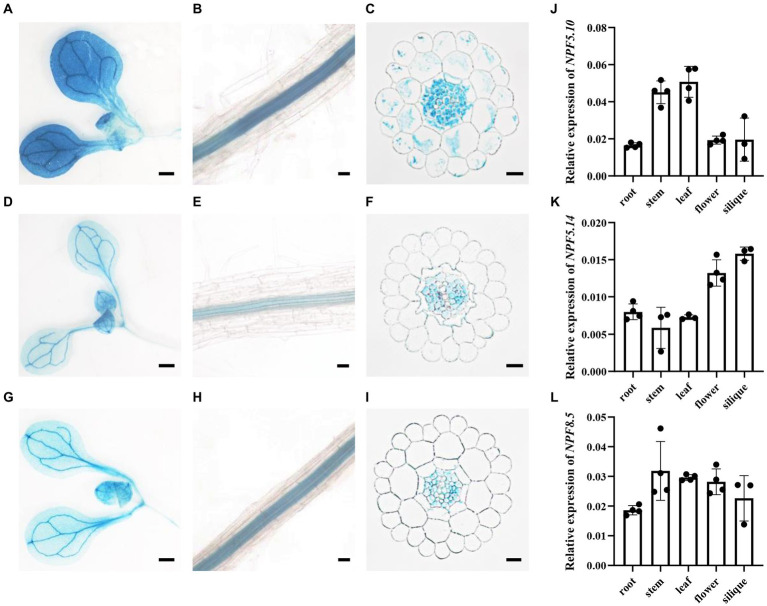
*NPF5.10*, *NPF5.14*, and *NPF8.5* are preferentially expressed in vascular tissues. Histochemical localization of GUS activity in *pNPF5.10: GUS* transgenic plants **(A–C)**, *pNPF5.14:GUS* transgenic plants **(D–F)** and *pNPF8.5:GUS* transgenic plants **(G–I)**. GUS staining of seedling shoots **(A,D,G)**, seedling roots **(B,E,H)** or cross-sectioned seedling roots **(C,F,I)**. Wild type (Col-0) were grown hydroponically for 28 days, the relative amounts of *NPF5.10* **(J)**, *NPF5.14* **(K)**, and *NPF8.5* **(L)** transcript levels in root, stem, leaf, flower, and silique were determined by quantitative RT-PCR using *AtActin2* as an internal control. Values are mean ± SD, *n* = 3–4. Scale bar, 50 μm.

The tissue-specific expression pattern of *NPF5.10*, *NPF5.14*, and *NPF8.5* was determined by histochemical assay using *pNPF5.10:GUS*, *pNPF5.14:GUS*, and *pNPF8.5:GUS* transgenic plants. *NPF5.10*, *NPF5.14*, and *NPF8.5* had similar expression patterns. In shoots, they were mainly expressed in leaf veins (Figures 3A,D,G). In roots, GUS activity was detected exclusively in root vascular stele (Figures 3B,E,H). To further reveal which cell type are *NPF5.10*, *NPF5.14*, and *NPF8.5* expressed, cross section of *pNPF5.10:GUS*, *pNPF5.14:GUS*, and *pNPF8.5:GUS* was examined. GUS activity was detected in pericycle and parenchyma cells (Figures 3C,F,I). After bolting, the expression patterns of *NPF5.10*, *NPF5.14*, and *NPF8.5* in different tissues were investigated by quantitative real-time PCR analysis (Figures 3J–L). The expression of *NPF5.10* was mainly expressed in stem and leaf, *NPF5.14* was mainly expressed in flower and silique, while *NPF8.5* was expressed consistently in all tissues.

### Overexpression of *NPF5.10*, *NPF5.14*, or *NPF8.5* reduced nitrate content in the vacuoles

**Figure 4:**
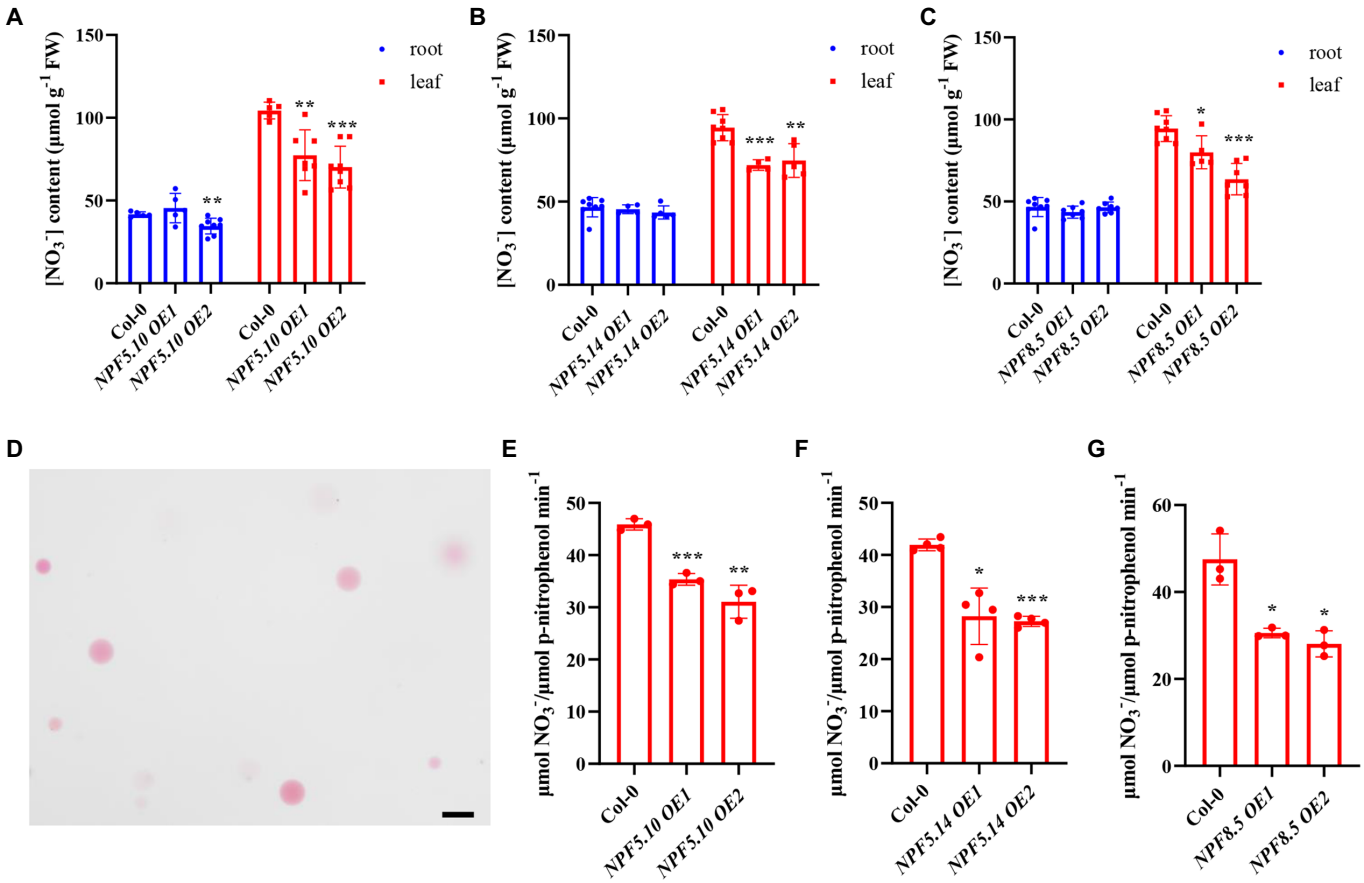
Vacuolar nitrate content reduced in overexpression lines. **(A–C)** Plants were grown hydroponically for 28 days, the leaves and roots of *NPF5.10*, *NPF5.14*, and *NPF8.5* overexpression lines were harvested to analyze nitrate contents by HPLC. Values are mean ± SD, *n* = 4–8. **(D)** Mesophyll protoplasts were isolated from leaves and then vacuoles were isolated from mesophyll protoplasts after 28 days of hydroponics, Scale bar, 50 μm. **(E–G)** Nitrate content in the vacuoles was determined by HPLC, acid phosphatase (ACP) activity specific to vacuole was determined and used to normalize nitrate accumulation. Values are mean ± SD, *n* = 3–4. Statistical significance was determined by Student’s *t*-test (^*^p < 0.05; ^**^p < 0.01; ^***^p < 0.001).

To further determine if NPF5.10, NPF5.14, and NPF8.5 indeed mediate vacuolar nitrate efflux in *A. thaliana*, we generated transgenic plants overexpressing *NPF5.10*, *NPF5.14*, and *NPF8.5* under the control of the 35S promoter (Supplementary Figure S4). HPLC analysis indicated that leaf nitrate content was lower in the overexpression lines than the wild-type control (Figures 4A–C). We also isolated intact vacuoles (Figure 4D) to directly determine vacuolar nitrate content, and the results showed that nitrate concentration was significantly lower in overexpression plant vacuoles than the wild types (Figures 4E–G). Together, these data consistently suggest that NPF5.10, NPF5.14, and NPF8.5 mediate nitrate uptake from vacuoles to cytosol, thus reallocating vacuolar nitrate when needed.

### Increased nitrate uptake activity in the sextuple mutant

**Figure 5:**
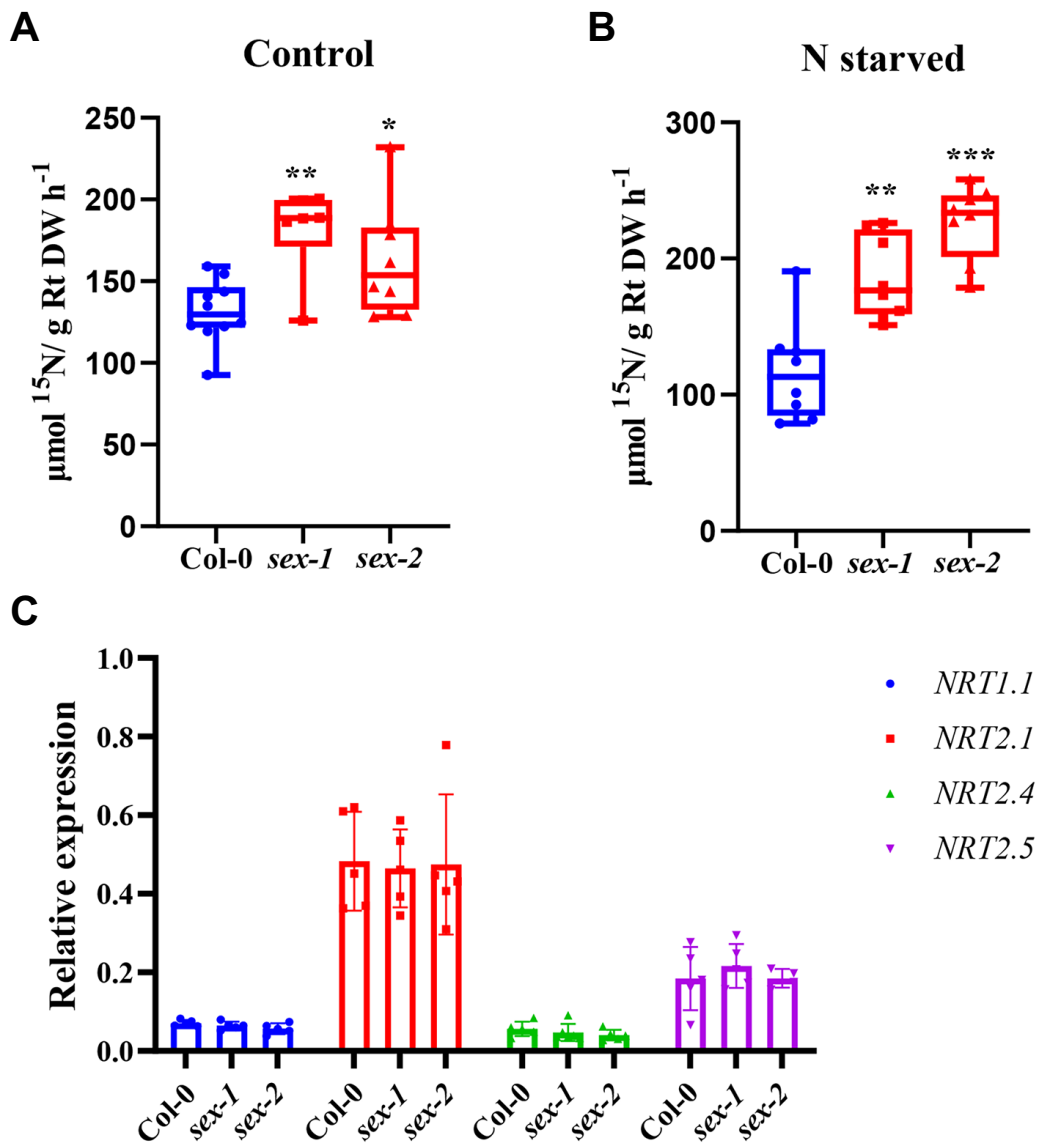
Nitrate uptake activity increased in sextuple mutant. **(A)** Plants were grown hydroponically for 21 days and treated with K^15^NO_3_ for 30 min. **(B)** Plants were grown hydroponically for 20 days and transferred to nitrate-starved nutrient solution for 1 day, then treated with K^15^NO_3_ for 30 min. Root uptake activity were determined. Box edges represent the 25th percentile, median, and 75th percentile of the data. Whiskers show the minimum and maximum. *n* = 6–10. **(C)** Expression of *NRT1.1*, *NRT2.1*, *NRT2.4*, and *NRT2.5* was determined by quantitative RT-PCR, and *AtActin2* was used as an internal control. Values are mean ± SD, *n* = 4–6. Statistical significance was determined by Student’s *t*-test (^*^p < 0.05; ^**^p < 0.01; ^***^p < 0.001).

Given that NPF5.10, NPF5.14, and NPF8.5 actively efflux nitrate from the vacuole, functional disruption of those transporters is expected to retain nitrate in vacuoles. However, our previous study did not observe any significant effect in the triple mutant *npf5.11 npf5.12 npf5.16*. To test if there is any functional redundancy, we then knocked out *NPF5.10*, *NPF5.14,* and *NPF8.5* using CRISPR/Cas9 and quadruple mutant (*npf5.11 npf5.12 npf5.16 npf5.10*), quintuple mutant (*npf5.11 npf5.12 npf5.16 npf5.10 npf5.14*), and sextuple mutant (*npf5.11 npf5.12 npf5.16 npf5.10 npf5.14 npf8.5*) were generated (Supplementary Figure S5A). We analyzed nitrate contents in leaves and roots of these mutants and wild type under both control and nitrogen starvation conditions (Supplementary Figures S5B–D), as well as nitrate contents in xylem sap (Supplementary Figure S6). However, still, no obvious difference was observed between the wild type and mutants. To our surprise, ^15^NO_3_^−^ uptake activity of the sextuple mutant lines was higher than that of the wild type (Figure 5), especially after 1 day of nitrogen deficiency treatment (Figure 5B). We then determined the expression of nitrate uptake transporter genes *NRT1.1*, *NRT2.1*, *NRT2.4*, and *NRT2.5* in wild-type and sextuple mutant exposed to nitrogen deficiency treatment, but no difference was observed (Figure 5C). We also noticed that the expression of *NPF5.10*, *NPF5.14*, *NPF8.5*, *NPF5.11*, *NPF5.12*, and *NPF5.16* was almost not inducible upon nitrogen deficiency (Supplementary Figure S7).

### Atclca participates in nitrate efflux from vacuoles under nitrogen deficiency

**Figure 6:**
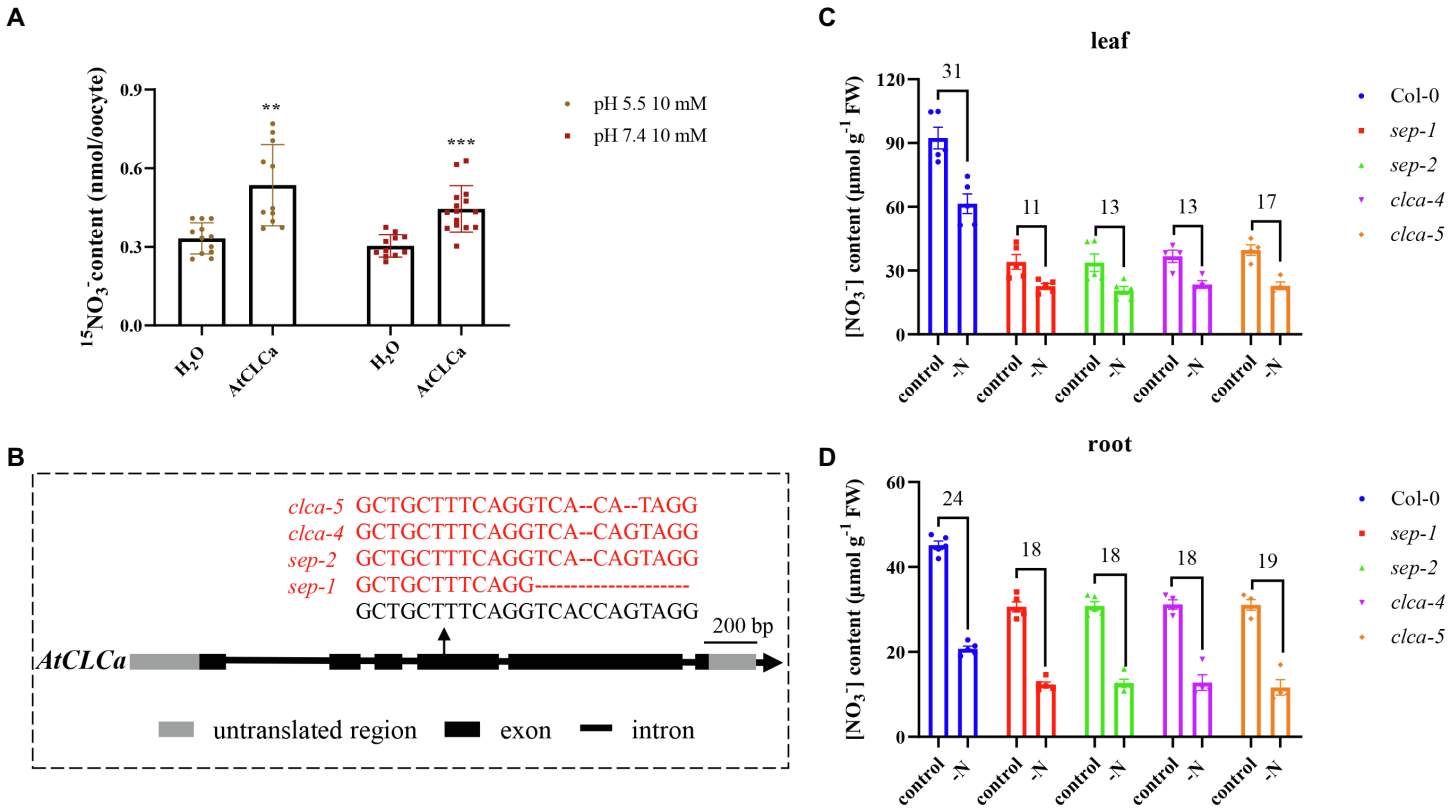
Nitrate content decreased in *clca* and the septuple mutant. **(A)** Oocytes injected with H_2_O, or cRNA of *AtCLCa* were incubated in ND96 solution for 2 days at 16°C. Oocytes were then transferred and incubated for 6 h in buffer containing 10 mM ^15^NO_3_^−^ with pH at 5.5 or 7.4. Values are mean ± SD, *n* = 11–15 oocytes. Statistical significance was determined by Student’s *t*-test (^**^p < 0.01; ^***^p < 0.001). **(B)** Schematic representation of the *AtCLCa* alleles in the *sep-1*, *sep-2*, *clca-4*, and *clca-5* mutants. Mutations were identified by RT-PCR and sequencing. **(C,D)** Plants were grown hydroponically to 27 days old and allowed further growth for 1 day with nitrate (control) or nitrate starvation (-N), then leaves and roots of Col-0, *sep-1*, *sep-2*, *clca-4*, and *clca-5* were sampled to determine nitrate concentration by HPLC. Nitrate deduction after 1 day treatment of nitrate starvation was calculated in leaves **(C)** or roots **(D)**. Values are mean ± SD, *n* = 4–5. Statistical significance was determined by Tukey’s test (*p* < 0.001).

Given disruption of 6 NPF transporters still did not give expected effect on vacuolar nitrate content, we suspected more redundant transporters might exist. AtCLCa is a member of CLC family in *Arabidopsis* and mediates vacuolar nitrate accumulation as a 2NO_3_^−^/1H^+^ antiporter (Geelen et al., 2000; De Angeli et al., 2006); therefore, it might get involved in regulating vacuolar nitrate efflux. To test this hypothesis, nitrate uptake assay was performed using ^15^NO_3_^−^. Compared with water-injected oocytes, the *AtCLCa* cRNA-injected oocytes showed significantly enhanced ^15^NO_3_^−^ uptake (Figure 6A), suggesting it might mediate vacuolar nitrate efflux to the cytosol in plants. We then generated a single mutant *clca* and a septuple mutant *npf5.11 npf5.12 npf5.16 npf5.10 npf5.14 npf8.5 clca* using CRISPR/Cas9 (Figure 6B). Lower nitrate accumulation was observed in the mutant than in the wild type (Figures 6C,D), consistent with the results in (De Angeli et al., 2006). Moreover, nitrate content in the mutant decreased less than in the wild type after 1 day of nitrogen deficiency, though no further difference was observed between *clca* and the septuple mutant (Figures 6C,D). These data indicate that nitrate efflux from vacuole is controlled by multiple genes, and *AtCLCa* might be the major one.

### More nitrate accumulated in sextuple mutant under osmotic stress

**Figure 7:**
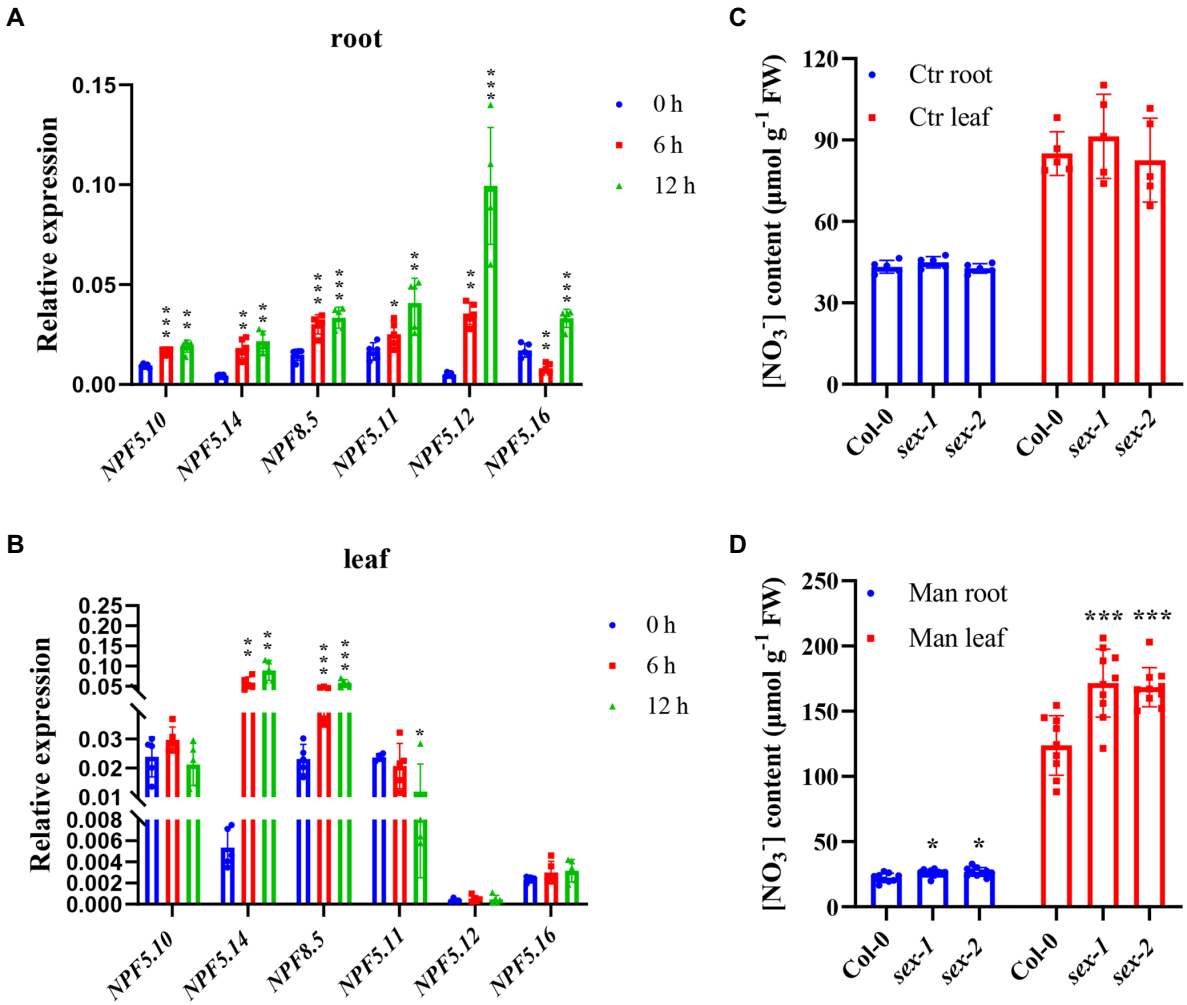
More nitrate accumulated in sextuple mutant under osmotic stress. **(A,B)** Wild type (Col-0) were grown hydroponically for 28 days, then treated with 300 mM mannitol for 0, 6, 12 h. Quantitative RT-PCR analysis of *NPF5.10*, *NPF5.14*, *NPF8.5*, *NPF5.11*, *NPF5.12*, and *NPF5.14* expression was performed. *AtActin2* was used as an internal control. Values are mean ± SD, *n* = 4–5. **(C,D)** Plants grown hydroponically for 28 days were incubated in hydroponic solution without (control) or with 300 mM mannitol (Man) for 12 h. Root and leave samples of Col-0, *sex-1,* and *sex-2* were harvested to analyze nitrate concentration by HPLC. Values are mean ± SD, *n* = 5 **(C)**, n = 9–10 **(D)**. Statistical significance was determined by Student’s *t*-test (^*^p < 0.05; ^**^p < 0.01; ^***^p < 0.001).

Given the sextuple mutant did not show any significant difference in nitrate content compared to the wild type, we therefore speculated that in addition to the proposed functional redundancy between those vacuolar nitrate transporters, it is also possible that vacuolar nitrate efflux occurs under certain circumstances. Previous studies proposed that vacuolar nitrate acts as an important osmotica regulator (Smirnoff and Stewart, 1985; Song et al., 2006; He et al., 2017; Wang et al., 2018), we then checked expression of *NPF5.10*, *NPF5.14*, *NPF8.5*, *NPF5.11*, *NPF5.12*, and *NPF5.16*. Interestingly, the results showed that all these genes were remarkably upregulated upon exposure to 300 mM mannitol, especially in the first 12 h (Figures 7A,B). Further analysis showed that nitrate contents in both leaves and roots were similar between the sextuple mutant and its wild-type control under normal condition (Figure 7C); however, when exposed to 300 mM mannitol treatment, nitrate content in the sextuple mutant was significantly higher than that in the wild type (Figure 7D). Given proline is an important osmolyte and *P5CS1* catalyzes the first step of Pro synthesis (Hu et al., 1992; Yoshiba et al., 1995), we then determined the *P5CS1* expression, but no significant difference was observed between the sextuple mutant and the wild type (Supplementary Figure S8A), indicating that proline biosynthesis might not get involved in the vacuolar nitrate-mediating osmotic stress response. Neither was enhanced tolerance to osmotic stress observed in the sextuple mutant compared to the wild type (Supplementary Figures S8B–F). These results suggest that vacuolar nitrate efflux is indeed facilitated by *NPF5.10*, *NPF5.14*, *NPF8.5*, *NPF5.11*, *NPF5.12*, and *NPF5.16* under osmotic stress, though the enhanced efflux might not contribute to enhanced osmotic stress tolerance.

## Discussion

The significance of vacuolar nitrate efflux is widely recognized though the underlying mechanism and exact function of this process remain elusive. Our current study identified that NPF5.10, NPF5.14, NPF8.5, and AtCLCa are functional nitrate transporters required for vacuolar nitrate efflux, in addition to those previously identified NPF5.11,NPF5.12, and NPF5.16 (He et al., 2017), and these functional redundant transporters facilitate vacuolar nitrate efflux under osmotic stress. These results suggest that vacuolar nitrate efflux is mediated by a very sophisticate mechanism.

Similar to NPF5.11, NPF5.12, and NPF5.16 (He et al., 2017), the NPF5.10, NPF5.14, and NPF8.5 proteins belong to the NRT1 family. And our data demonstrated that NPF5.10, NPF5.14, and NPF8.5 were tonoplast-localized nitrate transporters (Figures 1, 2; Supplementary Figure S1). And *NPF5.10*, *NPF5.11*, and *NPF8.5* were expressed in pericycle and xylem parenchyma cells (Figure 3). Thus, we proposed that NPF5.10, NPF5.14, and NPF8.5 together with NPF5.11, NPF5.12, and NPF5.16 regulate vacuolar nitrate efflux.

Consistent with our hypothesis, analysis of the nitrate content in *NPF5.10*, *NPF5.14*, and *NPF8.5* overexpression lines confirmed that NPF5.10, NPF5.14, and NPF8.5 indeed regulate the vacuolar nitrate efflux (Figure 4). However, there was no difference in nitrate accumulation between the quadruple, quintuple, sextuple mutant, and wild type under either nitrogen-sufficient or -starved conditions (Supplementary Figure S5). In addition, we found that ^15^NO_3_^−^ uptake activity of the sextuple mutant lines was higher than that of the wild type, especially after 1 day of nitrogen deficiency treatment (Figure 5). We speculate that the inhibition of vacuolar nitrate efflux in the sextuple mutant may cause the plant produce a nitrogen deficiency signal to absorb more nitrate. Given no transcriptional difference was observed for major nitrate uptake transporter genes, it is possible that protein level regulation might occur to those transporters in the sextuple mutant. Our data also indicates that there is serious redundancy between genes in regulating the vacuolar nitrate efflux process. In supporting of this hypothesis, AtCLCa might also get involved in regulating vacuolar nitrate efflux, as the septuple mutant (*npf5.11 npf5.12 npf5.16 npf5.10 npf5.14 npf8.5 clca*) showed significantly less nitrate reduction than the wild type after a day of nitrogen deficiency treatment, but no further decrease was observed when compared to *clca* (Figure 6). These data together suggest that vacuolar nitrate efflux under nitrogen deficiency is mediated by multiple genes, including *NPF5.11*, *NPF5.12*, *NPF5.16*, *NPF5.10*, *NPF5.14*, *NPF8.5*, and *AtCLCa*, among which *AtCLCa* might represent a major player.

In contrast, vacuolar sulfate and phosphate efflux appeared to be mediated by a much simpler mechanism, in which two major transporters were identified to be attributable and apparent nutritional role was proposed for either the vacuolar sulfate or vacuolar phosphate (Kataoka et al., 2004; Xu et al., 2019). Why there is such a huge difference between vacuolar sulfate/phosphate and nitrate efflux might remain an open question. However, in addition to the serious redundancy in controlling genes and the widely believed nutritional role of vacuolar nitrate, we instead propose that vacuolar nitrate efflux might occur preferentially or exclusively under certain environmental conditions other than nitrogen starvation. Previous studies have shown that vacuolar nitrate might act as osmolyte (Smirnoff and Stewart, 1985; Song et al., 2006; He et al., 2017; Wang et al., 2018). Our experimental results also favors this hypothesis, as we found that all the six genes (*NPF5.10*, *NPF5.14*, *NPF8.5*, *NPF5.11*, *NPF5.12*, and *NPF5.16*) were induced by mannitol (Figures 7A,B), and nitrate content in the sextuple mutant was significantly higher than that in the wild type under mannitol treatment (Figure 7D). Therefore, these data suggest that NPF5.10, NPF5.14, NPF8.5, NPF5.11, NPF5.12, and NPF5.16 regulate the vacuolar nitrate efflux mainly in response to osmotic stress rather than nutrient deficiency.

In conclusion, our research demonstrated that the mechanism of vacuolar nitrate efflux is highly complex, in contrast to the relatively straightforward mechanism of vacuolar sulfate and phosphate efflux. Serious genetic redundancy occurred in the regulation of vacuolar nitrate efflux and reallocation, and vacuolar nitrate efflux is conditionally inducible to play roles other than mere nutrient molecule, though the underlying mechanisms remain to be further determined.

## Data availability statement

The original contributions presented in the study are included in the article/Supplementary material; further inquiries can be directed to the corresponding author.

## Author contributions

J-MG and Y-TL conceived and designed the research. Y-TL performed most of the experiments. Y-TL, D-FL, T-TW, and S-YC contributed to the electrophysiological analysis. Z-JF and Y-TL measured nitrate contents. J-MG, Y-TL, and HL wrote the manuscript. All authors contributed to the article and approved the submitted version.

## Funding

This research was supported by the Strategic Priority Research Program of the Chinese Academy of Sciences (XDB27020101).

## Conflict of interest

The authors declare that the research was conducted in the absence of any commercial or financial relationships that could be construed as a potential conflict of interest.

## Publisher’s note

All claims expressed in this article are solely those of the authors and do not necessarily represent those of their affiliated organizations, or those of the publisher, the editors and the reviewers. Any product that may be evaluated in this article, or claim that may be made by its manufacturer, is not guaranteed or endorsed by the publisher.

## Footnotes

^1^ www.csbio.sjtu.edu.cn/bioinf/Cell-PLoc-2
