# Supplementary material for: Vacuolar nitrate efflux requires multiple functional redundant nitrate transporter in Arabidopsis thaliana
Source: Front Plant Sci. 2022 Jul 22;13:926809. doi: 10.3389/fpls.2022.926809 (PMC9355642; doi:10.3389/fpls.2022.926809)
Supplement: Supplementary file 2 [file Data_Sheet_1.pdf]

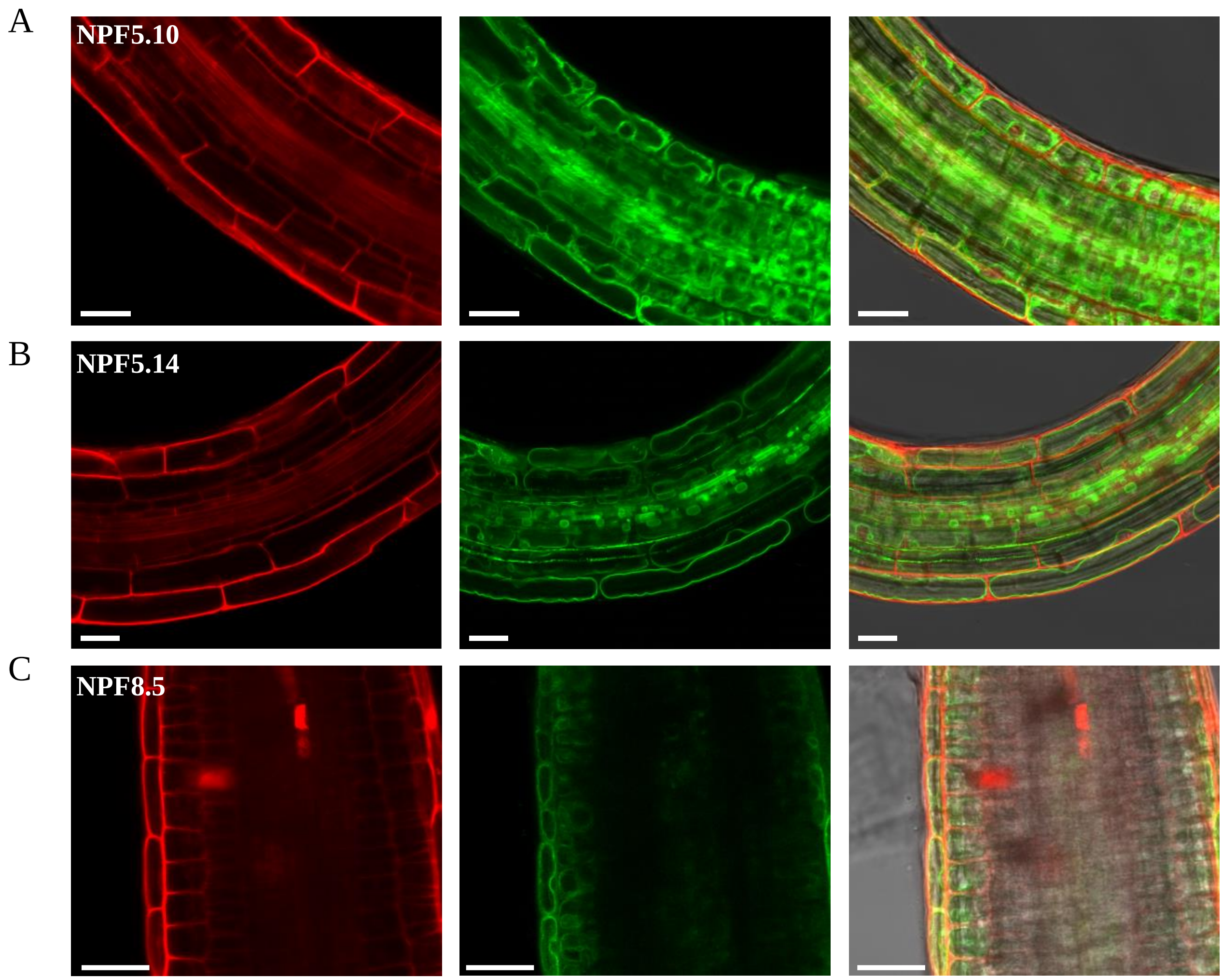

Figure S1 NPF5.10, NPF5.14 and NPF8.5 are located to the tonoplast. Expression of *p35S:NPF5.10-EGFP* **(A),** *p35S:NPF5.14-EGFP* **(B)** and *p35S:NPF8.5-EGFP* **(C)** in *Arabidopsis*. The green signals indicate EGFP, and the red signals indicate propidium iodide staining of the cell walls. Scale bar, 25 μm.

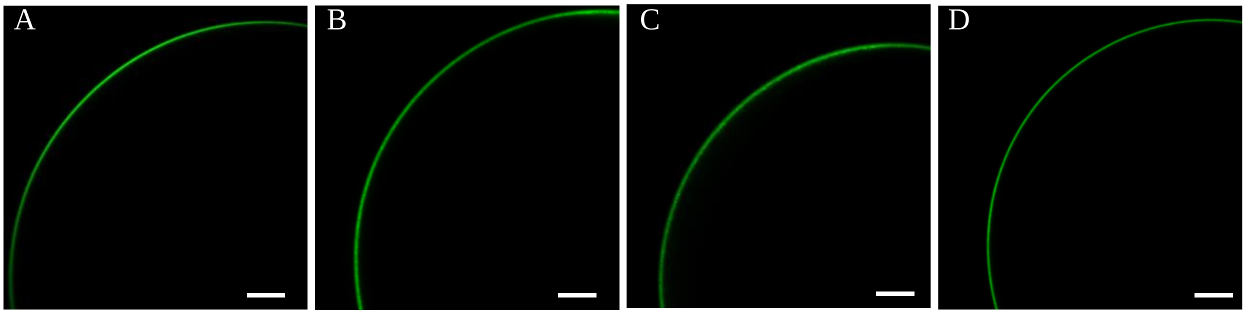

NPF7.2-EGFP NPF5.10-EYFP NPF5.14-EYFP NPF8.5-EGFP

Figure S2 NPF5.10, NPF5.14 and NPF8.5 expression in the oocyte cells. Oocytes injected with *p35S:NPF7.2-EGFP* **(A),** *p35S:NPF5.10-EYFP* **(B)**, *p35S:NPF5.14-EYFP* **(C)** and *p35S:NPF8.5-EGFP* cRNA **(D)** were imaged after incubation in ND96 solution for 2 days. Scale bar, 100 µm.

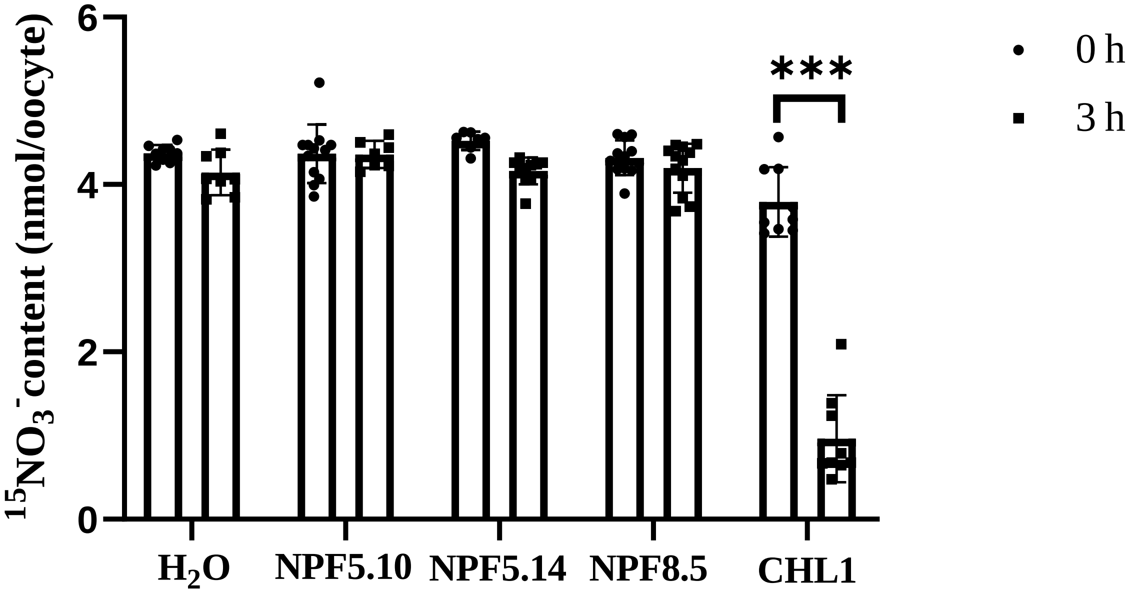

Figure S3 NPF5.10, NPF5.14 and NPF8.5 do not efflux nitrate across oocyte plasma membrane. Oocytes injected with H_2O, *NPF5.10*, *NPF5.14*, *NPF8.5* or *CHL1* cRNA were incubated in ND96 solution for 2 days at 16°C, then 50 nL of $K^{15}NO_3$ (100 mM) was injected into oocytes. At 0 h (immediately after injection) or after 3 h of incubation in ND96 solution at pH 5.5, oocytes were washed six times with ND96 solution and the ^{15}N contents were determined. Values are mean ± SD, n = 8–12 oocytes. Statistical significance was determined by Student's *t*-test (***$p < 0.001$).

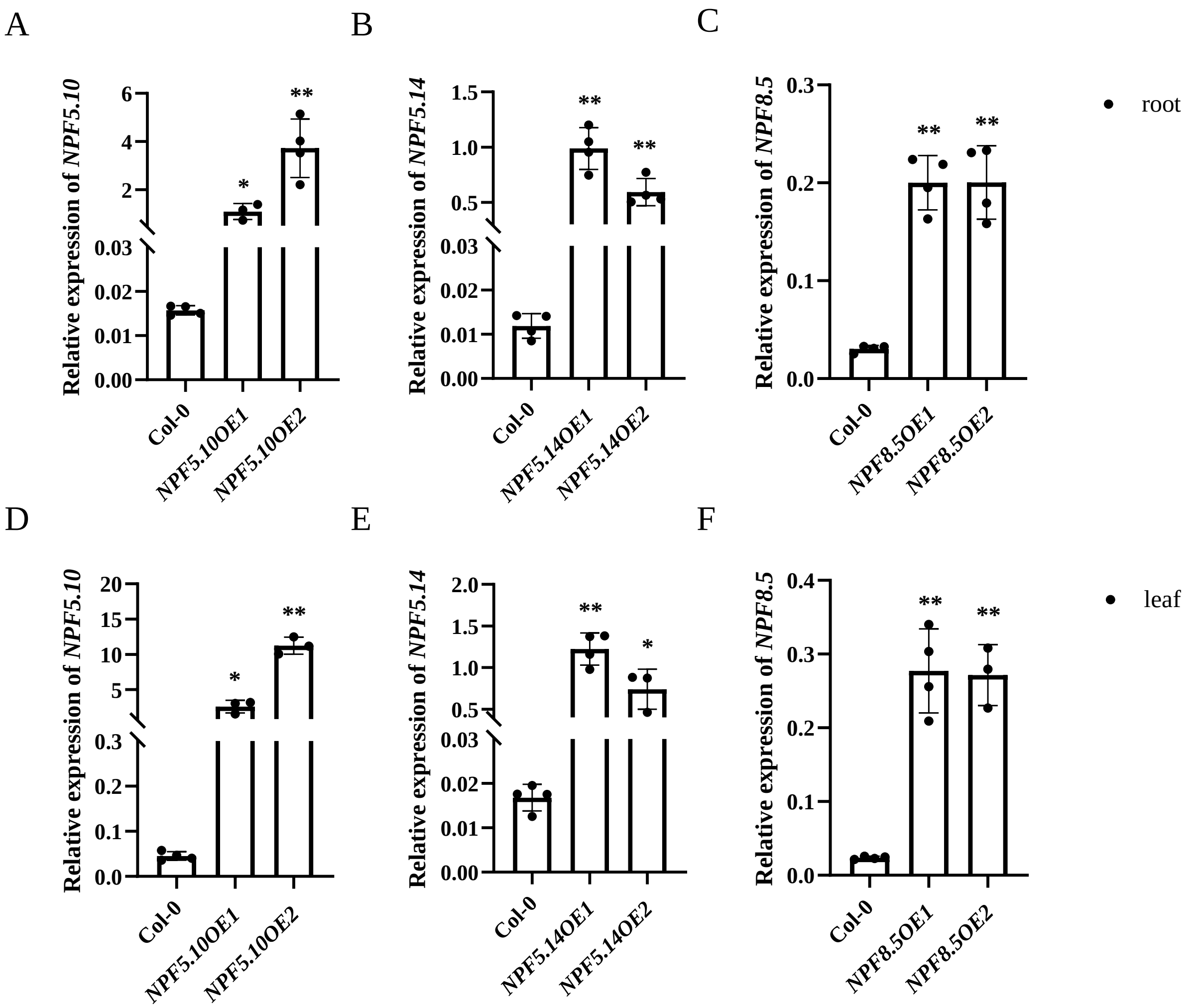

Figure S4 Expression levels of *NPF5.10*, *NPF5.14* and *NPF8.5* in their overexpression lines. Plants were grown hydroponically for 28 d, RNA was extracted from roots **(A-C)** and leaves **(D-F)** to identification of *NPF5.10*, *NPF5.14* and *NPF8.5* overexpression lines by qRT-PCR. Values are mean ± SD, n = 3-4. Statistical significance was determined by Student's *t*-test (*$p < 0.05$; **$p < 0.01$).

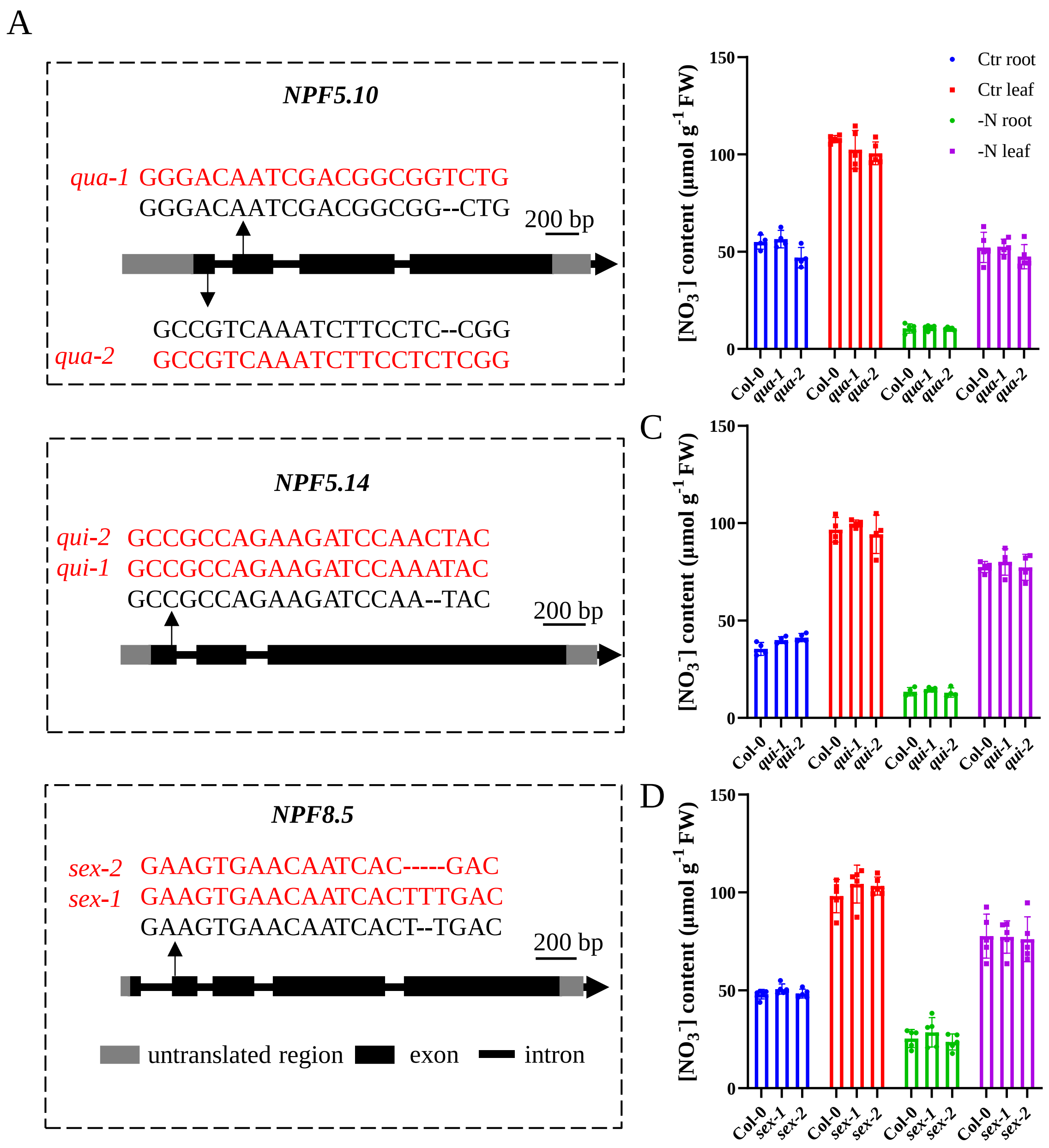

Figure S5 Nitrate content is not affected in the quadruple, quintuple or sextuple mutants. **(A)** Schematic representation of *qua-1*, *qua-2*, *qui-1*, *qui-2*, *sex-1*, *sex-2* and mutations were identification by RT-PCR. 27 days old plants grown hydroponically were kept growing (control) or treated with nitrate-starvation for 1 d (-N). Roots and leaves of Col-0, *qua-1* and *qua-2* **(B)**, Col-0, *qui-1* and *qui-2* **(C)**, Col-0, *sex-1* and *sex-2* **(D)** were harvested to analyze nitrate concentration by HPLC. Values are mean ± SD, n = 4-5.

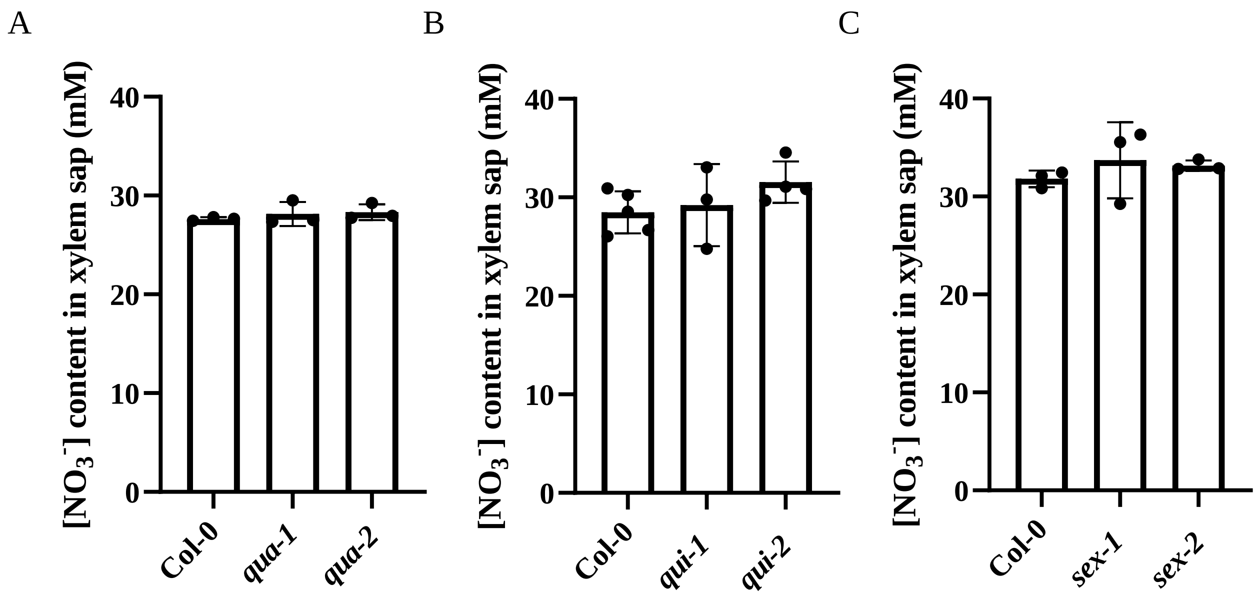

Figure S6 Root-to-shoot nitrate transport was not affected in the quadruple, quintuple or sextuple mutants. **(A)** quadruple mutant, **(B)** quintuple mutant, **(C)** sextuple mutant were grown hydroponically for 28 d, then the xylem sap was collected from floral stem cut for 2 h. Nitrate contents in xylem sap were analyzed by HPLC. Values are mean ± SD, n = 3-4.

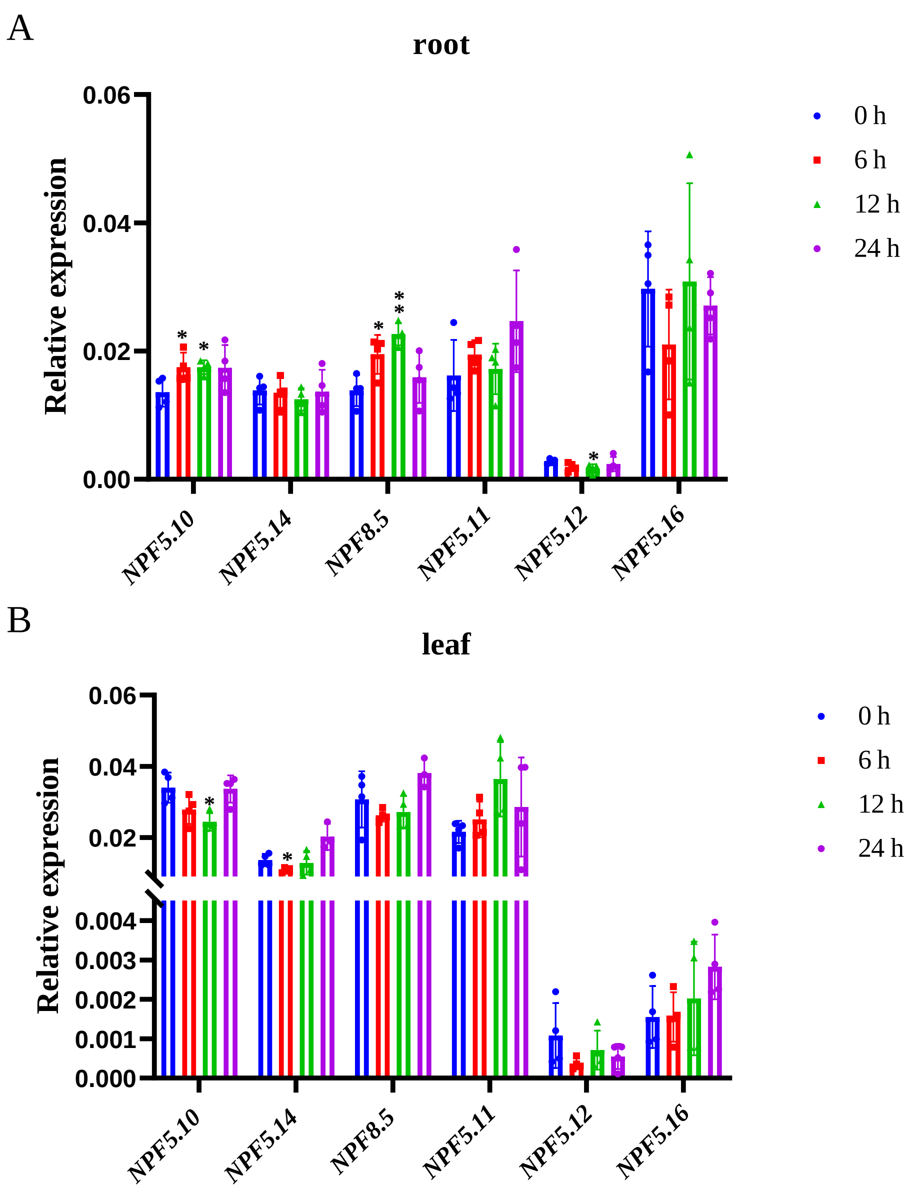

Figure S7 Expression of *NPF5.10*, *NPF5.14*, *NPF8.5*, *NPF5.11*, *NPF5.12* and *NPF5.16* is not inducible to nitrogen deficiency. Wild-type (Col-0) were grown hydroponically for 28 d, then were treated with nitrate-starved nutrient solution for0 h, 6 h, 12 h, 24 h. Quantitative RT-PCR determined the *NPF5.10*, *NPF5.14*, *NPF8.5*, *NPF5.11*, *NPF5.12* and *NPF5.14* transcript levels in root **(A)** and leaf **(B)** and *AtActin2* as used as an internal control. Values are mean ± SD, n = 3-4. Statistical significance was determined by Student's *t*-test (**p* < 0.05; ***p* < 0.01).

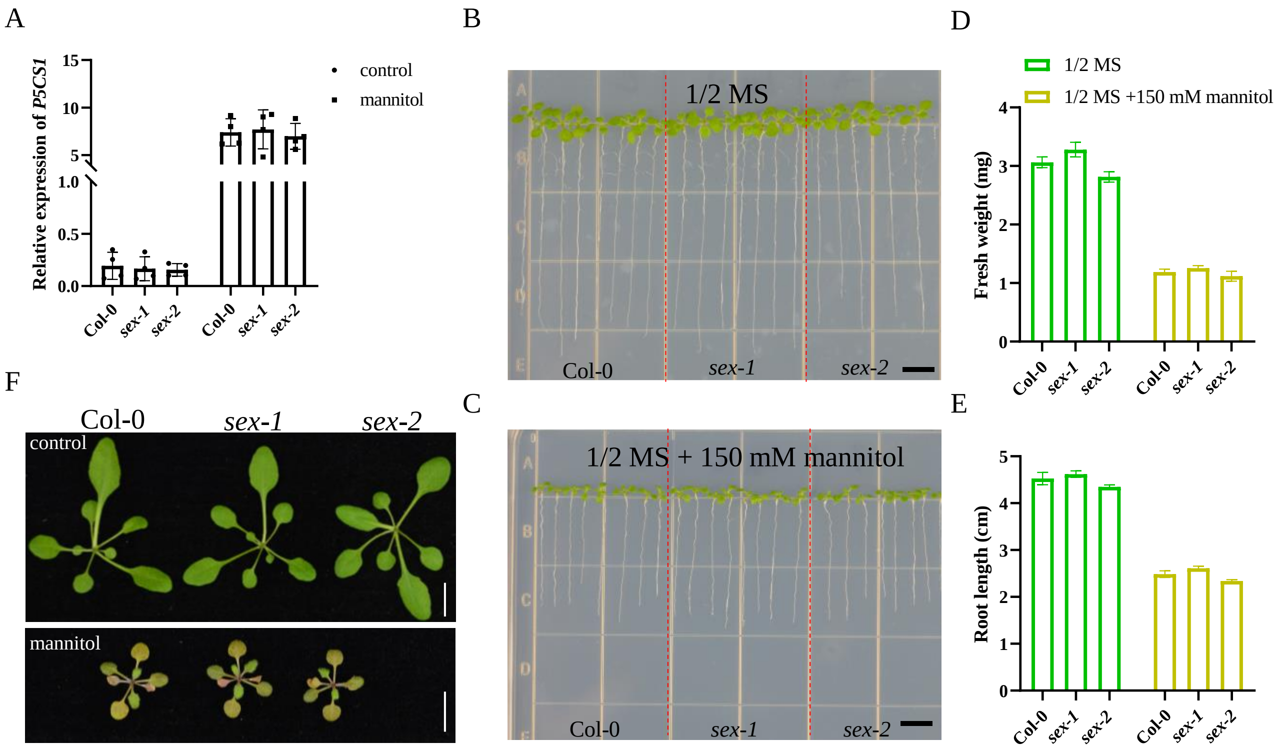

Figure S8. *P5CS1* expression and phenotypic assay under osmotic stress. **(A)** Plants were grown hydroponically to 28 d old before treatment with or without 300 mM mannitol for 12 h. Then the relative amounts of *P5CS1* transcript levels in Col-0, *sex-1* and *sex-2* leaves were determined by quantitative RT-PCR using *AtActin2* as an internal control. Values are mean ± SD, n = 4. **(B, C)** Plants were grown vertically on 1/2 MS plate with or without 150 mM mannitol for 7 d. Scale bar, 0.5 cm. **(D, E)** Fresh weight and root length in graph **(B, C).** Values are mean ± SE, n = 4. **(F)** Plants were grown in soil to 2 weeks of age and watered with hydroponic solution with or without 150 mM mannitol for 1 week. Scale bar, 1 cm.
